# GPR108, an NF-κB activator suppressed by TIRAP, negatively regulates TLR-triggered immune responses

**DOI:** 10.1371/journal.pone.0205303

**Published:** 2018-10-17

**Authors:** Danfeng Dong, Haisheng Zhou, Soon-Young Na, Rasma Niedra, Yibing Peng, Huajun Wang, Brian Seed, Guo Ling Zhou

**Affiliations:** 1 Center for Computational and Integrative Biology, Massachusetts General Hospital, Boston, Massachusetts, United States of America; 2 Department of Genetics, Harvard Medical School, Boston, Massachusetts, United States of America; 3 Department of Laboratory Medicine, Ruijin Hospital, Shanghai Jiaotong University School of Medicine, Shanghai, China; University of Tennessee Health Science Center, UNITED STATES

## Abstract

Higher vertebrates have evolved innate and adaptive immune systems to defend against foreign substances and pathogens. Sophisticated regulatory circuits are needed to avoid inappropriate immune responses and inflammation. GPR108 is a seven-transmembrane family protein that activates NF-κB strongly when overexpressed. Surprisingly, its action in a physiological context is that of an antagonist of Toll-like receptor (TLR)-mediated signaling. Cells from *Gpr108*-null mice exhibit enhanced cytokine secretion and NF-κB and IRF3 signaling, whereas *Gpr108*-null macrophages reconstituted with GPR108 exhibit blunted signaling. Co-expression of TLRs and GPR108 reduces NF-κB and IFNβ promoter activation compared to expression of either TLRs or GPR108 alone. Upon TLR stimulation GPR108 abundance increases and the protein engages TLRs and their partners to reduce MyD88 expression and interfere with its binding to TLR4 through blocking MyD88 ubiquitination. In turn GPR108 is antagonized by TIRAP, an adaptor protein for TLR and MyD88. The interrelationships between GPR108 and innate immune signaling components are multifactorial and point to a membrane-associated signaling structure of significant complexity.

## Introduction

A human GPR108 cDNA was initially identified among cDNAs enriched for expression in lung. It was found by sequence analysis to fall in the G-protein coupled receptor superfamily and was initially named lung seven transmembrane receptor 2 (LUSTR2)[1]. The encoded protein is predicted to bear an amino-terminal hydrophobic signal peptide sequence, a long extracellular domain, and a carboxy-terminal segment containing seven transmembrane domains. The sequence is highly conserved and phylogenetic counterparts can be found throughout the plant and animal kingdoms, in mammals, birds, insects, fish, arthropods, dipterans, nematodes, cress, rice and yeast[1]. The closest ortholog in mammals, *Gpr107*, shares 49% identity by amino acid sequence with *Gpr108* in mice.

GPR108 has been identified as a potent NF-κB activator in a cDNA screen [2]. In addition, it was also designated as one of the candidate genes for innate immunity in the Innate Illumina GoldenGate OPA Panel [3]. However, the function has remained unclear.

This study connects GPR108 with innate immunity through its action on signal transduction activated by Toll-like receptors (TLRs). Toll-like receptors are important innate immune receptors that initiate host defenses against microbial and viral pathogens. TLR signaling pathways depend on the adaptors MyD88 or TRIF (TIR domain-containing adaptor protein-inducing IFN-β), which induce proinflammatory cytokines and type I interferons [4–6]. As more has come to be understood about the TLR pathways, increasing attention has focused on negative regulators believed to attenuate the detrimental effects of inappropriate receptor engagement or the excessive production of cytokines and interferons [7–9]. The detailed mechanisms by which TLR signaling is modulated are not completely understood.

The present study demonstrates that GPR108 modulates immune responses initiated by TLRs through interactions with TLR adaptor protein MyD88 and TRAF6. *Gpr108* deficiency increases TLR-induced proinflammatory cytokine production in mouse embryonic fibroblasts (MEF) and macrophages. Reconstitution of *Gpr108*-null macrophages weakly activates NF-κB but reduces the level of NF-κB activation triggered by TLR inducers. In many settings, the forced expression of GPR108 is toxic. Transient expression of GPR108 in 293 cells elicits strong NF-κB and IFN-β promoter activation that is attenuated when TLR agonists are applied. Co-immunoprecipitation studies have shown that GPR108 can engage TLRs and the TLR-associated proteins TIRAP, MyD88 and TRAF6. A potential mechanism involves GPR108 competition with TLR4 to bind to MyD88 by modulating the E3 ligase TRAF6 which mediates ubiquitination of MyD88. In turn, the expression of GPR108 as an immune activator is restricted by TIRAP. Our results indicate that GPR108 may act to keep TLR-mediated immune responses within an appropriate range.

## Materials and methods

### Animals

Mouse studies were conducted under protocols approved by the institutional animal care and usage committee of Massachusetts General Hospital.

The targeting BAC strategy used to generate *Gpr108*-null mice has been previously described [10, 11]. The targeted clones MT1 and MT2 were screened by MLPA using multiplex MLPA probes (S1 Table). Chimeric mice were generated by injecting 8 to 12 targeted MT2 embryonic stem cells into C57BL/6 blastocysts. Five male chimeric animals were produced, three of which transmitted the *Gpr108* mutation into the next generation. Heterozygous mice were maintained, and mating was initiated to generate homozygous mutants. Genotyping primers P1-4 are shown in S1 Table.

### Plasmid construction

The full-length mouse (m) m*Gpr108*, m*TLR3*, *TLR4* and *TLR7*, MyD88, TRIF, TIRAP, TRAF6, TAK1, TAB2, and Nemo cDNA were inserted into expression vector pCMV-3xflag, pCMV-3xHA. *Gpr108* was also cloned into pEGFP-N1, pmcherry-N1 and the lentiviral vector pCSGW_cherry, pCSGW_EGFP. *Gpr108* mutants were inserted into expression vector pCMV-3xHA as well. MyD88, TRIF, TIRAP, TRAF6, TAK1, TAB2 and Nemo cDNA expression clones were from the E-library of Massachusetts General Hospital DNA Core Facility. GPR108 tet-one inducible expression system was constructed by following the manufacturer’s instructions (Clontech). pcDNA3-TLR3-CFP and pcDNA3-TLR9-YFP were a gift from Doug Golenbock (Addgene plasmid # 13641 and # 13642). TLR7 and TLR9 cDNA were inserted into pEGFP-N1 and pmcherry-N1, respectively.

### Reagents and antibodies

Lipopolysaccharide (LPS), and doxycycline (DOX) were purchased from Sigma. Poly (I:C), imiquimod, R848, CpGODN362 were obtained from InvivoGen. Antibodies used in this study were as follows: Anti-actin antibody, Sigma; Anti-FLAG_Dylight 680 and HA Epitope Tag Antibody_IRDye800, Rockland; anti-Myc (908805), (Biolegend), anti-Myd88 (D80F5), anti-phospho-IRF-3 (Ser396) (D6O1M), anti-IκBα (44D4), anti-phospho-IκBα (Ser32) (14D4), anti-TRF6 (D21G3), anti-TRIF, anti-ubiquitin, anti-p-Tyr, Cell Signaling Technologies; anti-flag M2 agarose, Sigma; anti-cherry, Anti-Giantin, anti-GM130 antibody, ABCAM; IRDye 680 and IRDye 800CW conjugated Goat anti-Mouse IgG, LI-COR Biosciences; Peroxidase AffiniPure Goat Anti-Rabbit IgG and peroxidase AffiniPure Goat Anti-Mouse IgG, Jackson Immuno Research; Mito-tracker and Lyso-tracker, Invitrogen.

### Derivation of cell lines from *Gpr108*^-/-^ mice

Primary mouse embryonic fibroblasts (MEF) were derived from 13.5-day fetuses and cultured in Iscove's Modified Dulbecco's Medium (IMDM) with 10% iron-supplemented calf serum, glutamine, non-essential amino acids and 10 μg/mL gentamycin. Macrophage cells were differentiated for 7–10 days from bone marrow of 6–8 week wild-type and *Gpr108*^*-/-*^ mice in the presence of M-CSF in Dulbecco's Modified Eagle Medium (DMEM), 10% iron-supplemented calf serum, glutamine and 10 μg/mL gentamicin. Three immortalized macrophage cells WT, KO6 and KO9 were generated by introducing SV40 large T-antigen into bone marrow derived macrophage cells. Two subsequent macrophage cell lines, *Gpr108* iRc (inducible reconstituted cells) 1–1 and 2–2 were established through stable expression of *Gpr108* cDNA under the control of the Tet-one system in KO9 cells. The expression of GPR108 could be induced by 1 μg/mL doxycycline for 48h.

### RNA extraction, RT-qPCR and RT-MLPA

Total RNA was extracted and purified with an RNeasy kit (Qiagen, Valencia, CA), and reverse-transcribed using an iScript cDNA Synthesis kit (Bio-Rad Laboratories, Hercules, CA). Quantitative PCR was performed using iQ SYBR Green Supermix in triplicate on Bio-Rad CFX384 Touch Real-Time PCR Detection System. Primer sequences are shown in S2 Table. RT-MLPA as described before[12] was performed to quantitatively measure *Gpr108* gene expression in different tissues. RT-MLPA probes for control genes, GAPDH, actin, HPRT, TBP and *Gpr108* are shown in S3 Table.

### Construction of *GPR108*^-/-^ THP-1 cells

A Crispr/Cas9 strategy was used for generating *GPR108*^*-/-*^ THP-1 cells [13]. Paired gRNAs flanking the deletion region were cloned into Cas9 viral vector and transduced into THP1 cells. Seven gRNAs were used for targeting two regions on *Gpr108* located in exons 1 and 13 (gRNA sequences shown in S4 Table). The deletion clones were screened by amplifying the deletion region by PCR. PCR primers are shown in S4 Table. Two alleles produced by deletion were further verified by measuring the mRNA level of *Gpr108* using RT-qPCR.

### mRNA sequencing and data analysis

Total RNA was extracted from MEF cells or macrophages using Trizol (Invitrogen) and purified using RNeasy columns (Qiagen). The sequencing library was created following the manufacturer’s instructions using an mRNA sequencing kit (Illumina). Sequencing was performed in MGH NGS core. A list of mouse mRNAs was downloaded from the UCSC Genome browser database (http://hgdownload.cse.ucsc.edu/downloads.html#mouse). Sequences were matched to the mRNA database using either Bowtie or BLAST. For each read, only the best match was kept. The number of reads that matched each mRNA was then counted. Assuming the total number of transcripts in each cell is on the order of 500,000 copies [14] and the read count is proportional to the transcript copy number and the length of the transcripts, the number of reads was then converted to an estimated transcript copy number.

### Transfection and transduction

For gene expression in mammalian cells, 293 cells were transfected by using Lipofectamine 2000 (Invitrogen) according to manufacturer’s procedure. BMDM cells were transduced by lentivirus infection, which was prepared by transfecting lentiviral expression vectors together with the packaging vectors VSVG and Gag-pol at a ratio of 10:1:10 in 293T cells. The medium supernatants containing virus particles were collected, concentrated and overlaid on BMDM cells with 8 μg/mL polybrene.

### NF-κB and IFNβ reporter assay

293 cells were transfected with 10 ng NF-κB or IFNβ firefly luciferase reporter and 250 ng activator or control vector together with 1 ng *Gaussia* luciferase vector per well in 96 well plates. After 48–72 hours, cell supernatants were sampled to determine *Gaussia* luciferase activity to normalize the transfection efficiency and cell lysates were used to determine the *Photinus* luciferase activity using the Bio-Glo Luciferase Assay System (Promega). For BMDM cells, cells were transduced with a lenti-virus based NF-κB reporter tagged with *Gaussia* luciferase. The *Gaussia* luciferase activity was assessed after 72 h post-infection. All experiments were performed in triplicate.

### Cell stimulation and cytokine detection

Cells were seeded in 12-well plates for cytokine detection. After stimulating with LPS (1 μg/ml or 100 ng/ml as indicated), poly (I:C) (5 μg/ml) or imiquimod (5 μg/ml) for the indicated times, the concentrations of IL-6 and TNF-α in the supernatants were measured by ELISA kits (eBiosciences). Cytokine gene expression was analyzed by RT-qPCR.

### Immunofluorescence

Transfected COS cells or Hela cells grown on glass cover slips were washed with PBS and fixed for 10 min with 4% (w/v) paraformaldehyde in PBS at room temperature or with 100% methanol at -20°C for 1 hour. Cells were rinsed 5 times with PBS. The cells were permeabilized with 0.3% Triton X-100 prepared in blocking solution (PBS containing 3% BSA and 5% v/v goat serum) for 30 min. Cells were stained with the indicated antibodies for 2 h at room temperature, followed by incubation with fluorescent-labeled secondary antibodies. Imaging was performed on a Leica SP5 AOBS confocal microscope equipped with argon-krypton, and 543/594 nm helium-neon lasers. Images were acquired using a 40x or 63x objective and the appropriate filter combination.

### Co-IP and immunoblot analysis

For co-IP, cell lysates were obtained by incubating cells in lysis buffer (Tris pH 7.4 10 mM, NaCl 100 mM, EDTA 1 mM, EGTA 1 mM, Triton X-100 1%, glycerol 10%, SDS 0.1%, deoxycholate 0.5%, freshly added PMSF and protease inhibitor) for 20 min on ice, and were centrifuged at 12000 rpm for 10 min at 4°C. Supernatants were collected and incubated with anti-flag M2 agarose beads for 2 h at 4°C. After washing with cell lysis buffer three times, the binding protein was eluted with sample loading buffer followed by incubation for 10 min at 100°C. For immunoblot analysis, protein samples were subjected to SDS-PAGE and transferred to PVDF membranes (Millipore). Membranes were blocked with blocking buffer (5% milk PBST buffer or 3% BSA PBST) and further incubated with the indicated antibodies. β-actin was used as an internal normalization control. Protein bands were detected either by chemiluminescence or an Odyssey Infrared Imaging System.

### In vitro ubiquitination assays

For blots with tagged ubiquitin, cells were collected and follow the procedure which was described before[15].

### Statistical analyses

Student's t -test was used to determine the statistical significance of differences between groups.

## Results

### Generation of *Gpr108*^-/-^ mice

**Fig 1:**
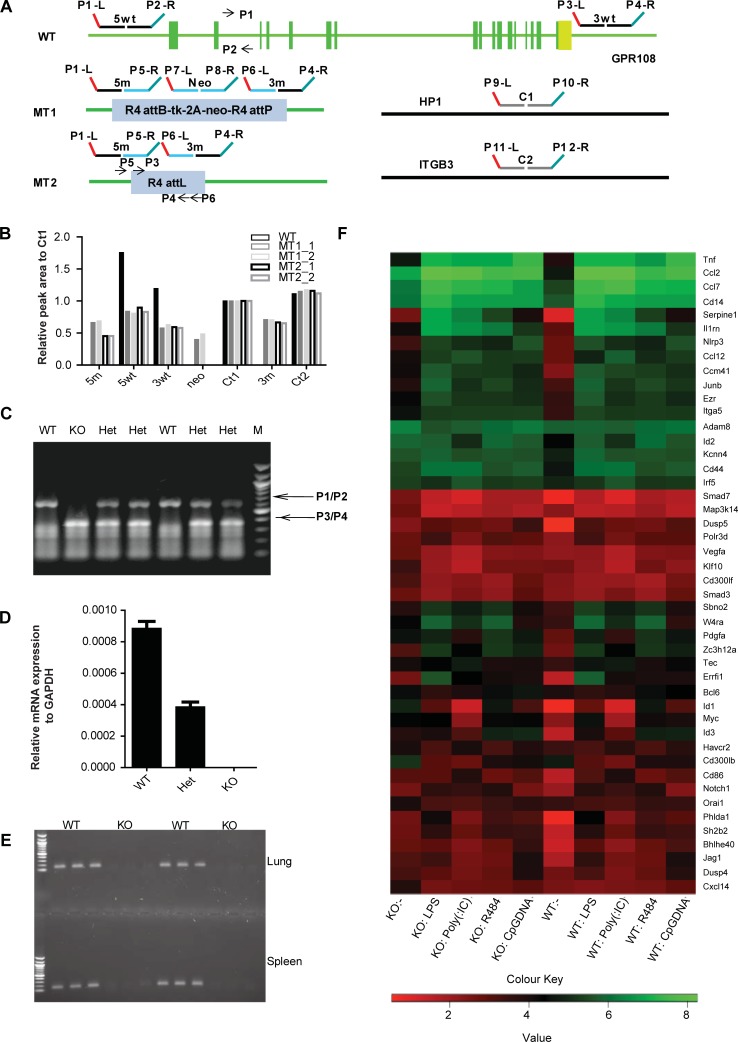
Generation of *Gpr108*-null mice. (**A**) Schematic diagram of construction of *Gpr108* knockout mice. The murine *Gpr108* locus contains 17 exons. Using a targeting BAC bearing the selection marker tk-2A-neo flanked by R4 integrase attB and attP sites, exons 1 to 17 were deleted, yielding the MT1 ES cell line. After expression of R4 integrase in MT1 cells, the resistance elements were removed as shown in MT2. The probes used for MLPA, 5wt, 3wt, 5m, 3m, neo, C1 and C2 are indicated. The primers P1/P2, P3/P4 used for PCR genotyping and P5/P6 for R4 deletion test are also shown. (**B**) Representative MLPA fragment analysis of 5wt, 3wt, 5m, 3m, neo, C1, C2 for different target events in wild-type cells as well as MT1 and MT2 targeted ES cells. Using HP1 (C1) and ITGB3 (C2) as internal controls to normalize intensities, the 5wt and 3wt signal intensities were observed to be diminished by approximately one half in correctly targeted MT1 cells compared to those in wt cells and the copy numbers of 5m, 3m and neo were identified as single copy or greater by analysis of all ES clones exhibiting resistance to G-418. In MT2 ES cells, the neo MLPA signal was not observed following excision by R4 integrase. (**C**) Mouse genotypes by PCR of wild-type (WT), heterozygous (Het) and homozygous (KO) mice are indicated. (**D**) *Gpr108* mRNA abundance in WT, Het and KO MEF cells. *Gpr108* mRNA was absent from KO MEF cells and present in Het at approximately 50% of the level found in wild-type MEF cells. (**E**) *Gpr108* mRNA detection in spleen and lung of WT and KO mice. *Gpr108* mRNA was absent in tissues from KO mice. (**F**). mRNA transcriptome panel of immune response and immediate early genes (IEGs) in BMDM cells derived from *Gpr108*^*+/+*^ (n = 3) and *Gpr108*^*-/-*^ (n = 3) mice in the absence or presence of different TLR agonists. Each column presents the mRNA expression of BMDM cells without or with different treatments. The intensity represents the magnitude of the difference. Red and green denote low and high expression, respectively.

The *Gpr108* locus was deleted in embryonic stem cells by a BAC-mediated homologous recombination method [11, 16]. The murine gene of 17 exons together with a 2 kb upstream region was replaced by a neomycin cassette as illustrated in Fig 1A. Correctly targeted clones (MT1) were identified by multiplex ligation-dependent probe amplification (MLPA) using the probes indicated in Fig 1A and 1B [17]. The neomycin cassette was flanked by R4 integrase attB and attP sites and was deleted by transient expression of R4 integrase in correctly targeted clones (MT2). Selected clones were injected into mouse blastocysts to produce chimeric mice. The heterozygous progeny of chimeric animals and homozygous progeny of heterozygous animals were identified by genomic PCR (Fig 1C) and confirmed by MLPA.

Mice heterozygous for *Gpr108* were mated to generate F2 offspring. From these matings, homozygous *Gpr108*-null offspring were viable and fertile and exhibited normal Mendelian segregation. Total RNA was prepared, and reverse transcription and qPCR were performed to analyze *Gpr108* gene expression in mouse embryonic fibroblast (MEF) cells. The results indicated that the homozygous MEF cells contained no *Gpr108* mRNA and that heterozygous MEF cells showed approximately half the transcript abundance found in wild-type MEF cells (Fig 1D). There was no *Gpr108* mRNA detected in the adult lung and spleen tissue of homozygous mice (Fig 1E). The levels of *Gpr108* mRNA in eleven adult tissues by RT-qPCR and RT-MLPA (two probes used) were similar whereas higher expression was detected in spleen, lung, stomach, large and small intestine and thymus compared to that in brain, heart, muscle and kidney (Panel A-C in S1 Fig). However, the signal of the endogenous GPR108 protein expression across different tissues is not detectable by using the different anti-GPR108 antibodies which may be due to its low level of expression.

### Enhanced TLRs-triggered immune responses and signaling in *Gpr108*-null cells

To study the phenotype of *Gpr108*–null mice, *Gpr108*^+/+^ and *Gpr108*^*-/-*^ E13.5 MEF cells were subjected to sequence-based transcript abundance analysis. The results showed that the expression of TLRs was elevated in *Gpr108*^-/-^ MEF cells (S5 Table), implying some connection between GPR108 and TLRs.

**Fig 2:**
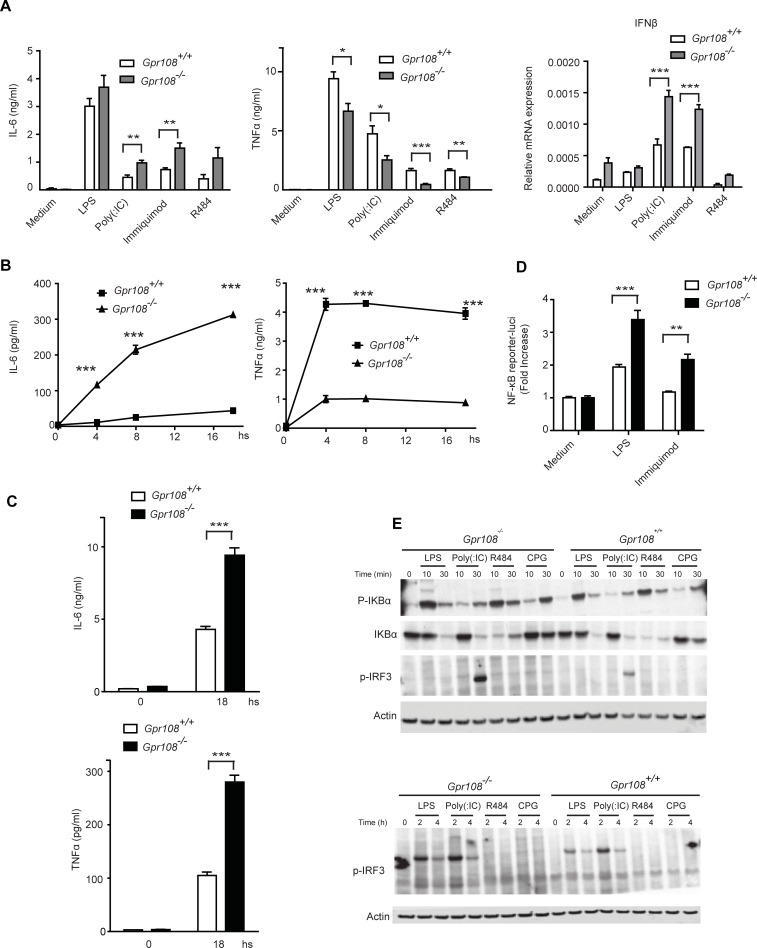
TLRs-triggered immune response was enhanced in *Gpr108*-null cells. (**A**) Primary macrophage cells derived from litter mated *Gp108*^+/+^ and *Gpr108*^-/-^ mice were treated with LPS (1 μg/ml, TLR4), Poly (I:C) (5 μg/ml, TLR3), immiquimod (50 μg/ml, TLR7) and R484 (10 μg/ml, TLR7). IL-6 and TNFα production were measured by ELISA after 18 hours treatment. The expression of IFNβ was determined by RT-qPCR after 8 hours treatment. (**B**) IL-6 and TNFα production were measured in immortalized *Gpr108*^+/+^ and *Gpr108*^-/-^ macrophages treated with LPS (100 ng/ml) at different time points. (**C**) IL-6 and TNFα production were measured in primary *Gpr108*^+/+^ and *Gpr108*^-/-^ MEF cells treated with LPS (100 ng/ml) for 18 hours. (**D**) iBMDM cells were transduced with a lenti-virus NF-κB reporter containing luciferase and treated with LPS (100 ng/ml) and immiquimod (50 μg/ml) for 18 hours. The luciferase activities were measured as fold increase by normalizing to the untreated cells as 1. (**E**) BMDM cells were stimulated with LPS (100 ng/ml), Poly (I:C) (5 μg/ml), R484 (10 μg/ml) and CPG (1μM) at different time points. The phosphorylated IRF3, IKBα and phosphorylated IKBα were measured by immunoblotting analysis with actin protein as its loading control. Data were shown as mean±SD (A-D). *p < 0.05, **p < 0.01, ***p<0.001. All experiments were repeated for at least three times.

Cytokine secretion and interferon expression were tested in *Gpr108*^*+/+*^ and *Gpr108*^*-/-*^ bone marrow-derived macrophage cells (BMDM) exposed to TLR4 (lipopolysaccharide, LPS), TLR3 (Poly (I:C) or TLR7 (imiquimod, R848) agonists. mRNA profiling was conducted on treated or untreated cells. The up-regulated genes which are selected on the right side of heatmap in *Gpr108*^-/-^ macrophage cells were found to significantly overlap those up-regulated in the TLR agonist-treated cells suggesting the existence of a constitutive inflammatory status in *Gpr108*^-/-^ macrophage cells (Panel D in S1 Fig**)**. Approximately fifty immune-related genes and immediate early genes (IEGs) up-regulated in naïve *Gpr108*^-/-^ macrophage cells and activated macrophage cells as examples were clustered shown in Fig 1F. Following stimulation, little difference in transcript abundance between activated wild type and knockout cells could be identified (Fig 1F and Panel D in S1 Fig). However, in protein level BMDM cells from *Gpr108*^-/-^ mice produced a higher level of proinflammatory cytokine IL-6 and a lower level of TNFα compared to cells from *Gpr108*^*+/+*^ mice (Fig 2A). This phenomenon might represent the downstream inhibitory action of IL-6 on TNFα production upon LPS stimulation in BMDM cells, which is also observed in other studies before [18]. In addition, higher level of IFNβ transcripts was detected in *Gpr108*^-/-^ BMDM cells than that in *Gpr108*^+/+^ BMDM using quantitative RT-PCR (Fig 2A). In later study, *Gpr108*^+/+^ and *Gpr108*^-/-^ BMDM cells were immortalized by expression of SV40 large T antigen [19]. A time course study of cytokine production in iBMDM (immortalized BMDM) cells and showed enhanced IL-6 and decreased TNFα secretion in LPS-stimulated *Gpr108*^-/-^ iBMDM cells, consistent with the observations in primary cells (Fig 2B). Previous study has showed that MEFs expressed high levels of mRNA for TLR1, TLR2, TLR3, TLR4, TLR5, TLR6, TLR7, TLR8 and TLR9 and MEFs were highly responsive to TLR-ligand activation [20]. So MEF cells were tested for LPS treatment as well. However, in *Gpr108*^-/-^ MEF cells, LPS stimulation led to higher levels of both IL-6 and TNFα (Fig 2C). Higher level of TNFα and IL-1β but not IL-6 transcripts was also observed in *Gpr108*^-/-^ THP1 cells which were generated by Crispr/Cas9 gene editing (Panel A and B in S2 Fig)[21]. These data showed GPR108 deficiency would result in a constitutive low level of inflammatory status and the enhanced LPS-triggered immune responses in cells, but also showed some discrepancy on cytokine secretion in different types of cells, implying regulation complexity of GPR108 in distinct types of cells.

In addition, the signal proteins were tested in BMDM cells. *Gpr108*^-/-^ cells generated a significantly stronger NF-κB response than *Gpr108*^+/+^ cells following LPS or imiquimod stimulation (Fig 2D). Activation of the NF-κB pathway (measured by phosphorylated IκBα) and the IRF3 pathway (measured by phosphorylated IRF3) was greater in TLR-stimulated *Gpr108*^-/-^ BMDM cells than in *Gpr108*^+/+^ cells, even though phosphorylated IRF-3 could only be detected in response to LPS and Poly (I:C) (Fig 2E). These findings confirmed the view that *Gpr108* deficiency enhances TLR-triggered proinflammatory signaling in BMDM cells.

To further explore the role of GPR108 in TLR-triggered immune responses, we performed an LPS lethal challenge of *Gpr108*^-/-^ mice. All the *Gpr108*^-/-^ mice died within 10 hours, while 21% of wild-type mice remained alive at the endpoint, indicating that *Gpr108*^-/-^ mice are more susceptible to LPS challenge (Panel C in S2 Fig). The serum level of a series of cytokines were tested and only IL-6 was found to be slightly elevated in *Gpr108*^-/-^ mice, but statistical significance was not reached (Panel D in S2 Fig). Given the observed mild phenotype in *Gpr108*^-/-^ mice, it was predicted that GPR108 might tune TLR signaling in certain specific cells, but its regulation might be hidden by other redundant regulators in animal level.

### GPR108 acts as an activator itself but limits TLR-triggered immune responses in GPR108-induced BMDM cells and 293 cells

**Fig 3:**
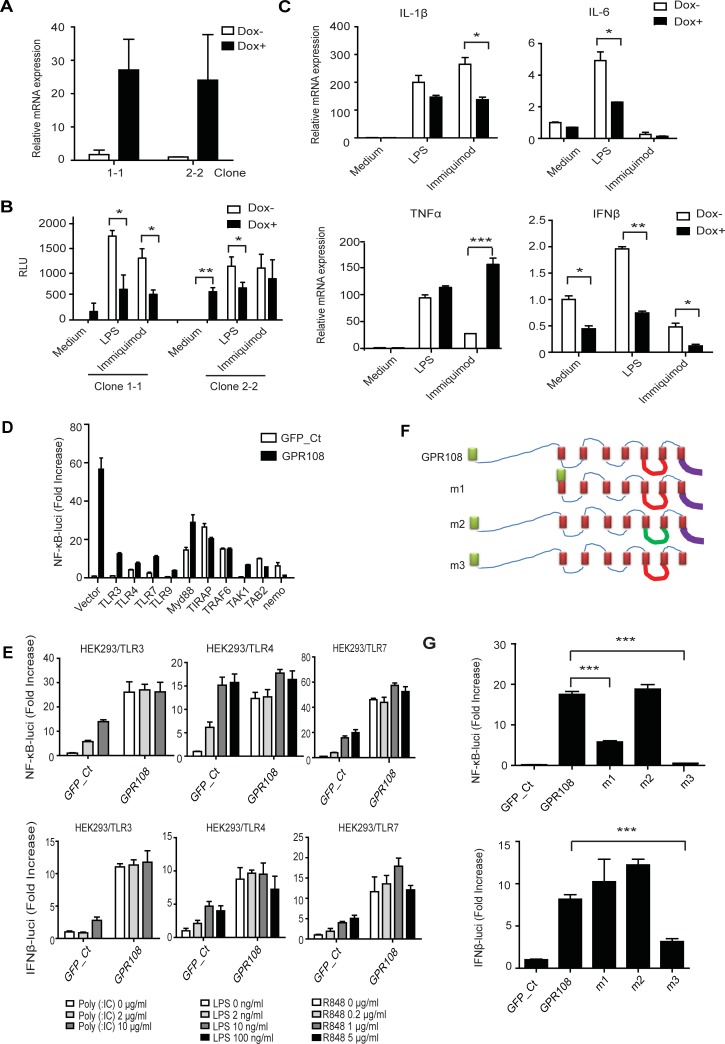
GPR108 works as an activator but limits TLRs-triggered NF-κB and IFNβ response. (**A**) Two *Gpr108*-inducible clones 1–1 and 2–2, derived from *Gpr108*^-/-^ iBMDM cells were stimulated with Dox (1μg/ml) for 48 hours. The expressions of *Gpr108* were determined by RT-qPCR with actin gene as an internal control. (**B**) Two *Gpr108*-inducible clones 1–1 and 2–2 were transduced with NF-κB reporter containing luciferase. Cells were pretreated with or without Dox for 48 hours and then the luciferase activities were measured after the stimulation with LPS (100 ng/ml) and immiquimod (50 μg/ml) for 18 hours. (**C**) Gene expressions of IL-1β, IL-6, TNFα and IFNβ in DOX treated or untreated cells (clone1-1) (48 hours) were analyzed after exposure to LPS (100ng/ml) and immiquimod (50 μg/ml) for 1.5 hours. Actin was used as an internal control. (**D**) HEK293 cells were transfected with NF-κB-luciferase reporter and Flag-vector or Flag-TLRs, together with GFP or GPR108 plasmids. The NF-κB luciferase activities (fold increase) were analyzed after 48 hours transfection. (**E**) HEK293/TLR3, TLR4 and TLR7 stable cell lines were transfected with NF-κB-luciferase or IFNβ-luciferase reporter, together with control vector or *Gpr108*. Cells were treated with their corresponding agonists Poly (I:C) (TLR3), LPS (TLR4) and R484 (TLR7) at different doses for 18 hours and were analyzed for luciferase activities (fold increase). (**F**) Schematic diagram of the *Gpr108* mutants: m1, an N-terminal domain deletion mutant with a leader peptide (green box); m2, loop3 replacement (AVPFQ (red line) was replaced with GGGGS (green line)); m3, a C-terminal domain deletion (purple line). (**G**) HEK293 cells were transfected with NF-κB-luciferase reporter, together with HA-vector, *Gpr108* or *Gpr108* mutants. The luciferase activities (fold increase) were analyzed after 48 hours transfection. Data were shown as mean±SD.*p < 0.05, **p < 0.01, ***p<0.001. All the experiments were repeated for at least three times.

The tempt to generate the GPR108-overexpressed stable cells in different type of cells totally failed, which may mean that higher level of expression of GPR108 is toxic to the cells. Therefore, a tetracycline-based inducible expression system was used to reconstitute GPR108 expression in *Gpr108*-null immortalized macrophages. Two clones 1–1, 2–2 were identified by puromycin selection. After exposure to doxycycline (DOX) for 48 hours, both clones had a greatly increased mRNA expression of *Gpr108* (Fig 3A), but the increase in protein level was imperceptible. To further study the effect of GPR108 on TLRs-triggered signaling, an NF-κB reporter expressing luciferase was transduced into iBMDM cells. Following introduction of an NF-κB reporter, weak luciferase activity was observed in both clones after 48 hours of exposure to doxycycline, recapitulating the later results of overexpression studies that showed GPR108 itself can act as an NF-κB activator (Fig 3B). However, induction by doxycycline greatly reduced NF-κB activation through LPS and imiquimod stimulation (Fig 3B). The expression of IL-1β, IL-6 and IFNβ mRNAs was decreased but the expression of TNFα mRNA was increased following induction which repeated the previous observation in *Gpr108*-*null* and wild-type BMDM cells.

Transient overexpression of GPR108 in 293 cells induces strong NF-κB reporter activity (Fig 3D). However, when GPR108 and TLR3, 4, 7 or 9 were co-expressed in 293, NF-κB reporter activity was greatly reduced. In addition, co-expression of GPR108 with the downstream proteins MyD88, TIRAP, TRAF6, TAK1, TAB2 and Nemo resulted in lower NF-κB activity compared to expression of GPR108 alone (Fig 3D). To further explore this, 293-TLR4, TLR3 and TLR7 stable cell lines were treated with the TLR agonists LPS, Poly (I:C) and R848, respectively. In the absence of GPR108, all agonists could induce NF-κB and IFNβ reporter activity in a dose-dependent manner (Fig 3E). When GPR108 was expressed in these TLR stable cell lines, high NF-κB and IFNβ reporter activities were observed without stimulation but no obvious dose-dependent trend and addition activation was seen (Fig 3E), which indicates the dual functions of GPR108, not only NFkB and IFNβ activation but also its negative effect on TLRs-triggered stimulation. We can artificially transiently overexpress GPR108 to see its dominant strong activation, but in fact GPR108 cannot be physiologically highly and stably expressed in cells due to its intolerance and its repelled low expression are sufficiently control TLR-triggered immune response in proper range.

GPR108 has a long N-terminal extracellular domain, three extracellular loops, three intracellular loops and a short C-terminal intracellular domain (Fig 3F). Three mutants, m1, m2 and m3 were constructed (Fig 3F). The N-terminal and C-terminal domains were truncated to give mutants m1 and m3, respectively. Internal loop 3 was replaced by a substitute linker sequence in mutant m2 (Fig 3F). C-terminal deletion m3 completely abolished NF-κB and IFNβ activation, demonstrating the importance of the C-terminal domain for signal transduction. The m1 deletion affected NF-κB but not IFNβ activity (Fig 3G).

### GPR108 engages TLRs and its partners

**Fig 4:**
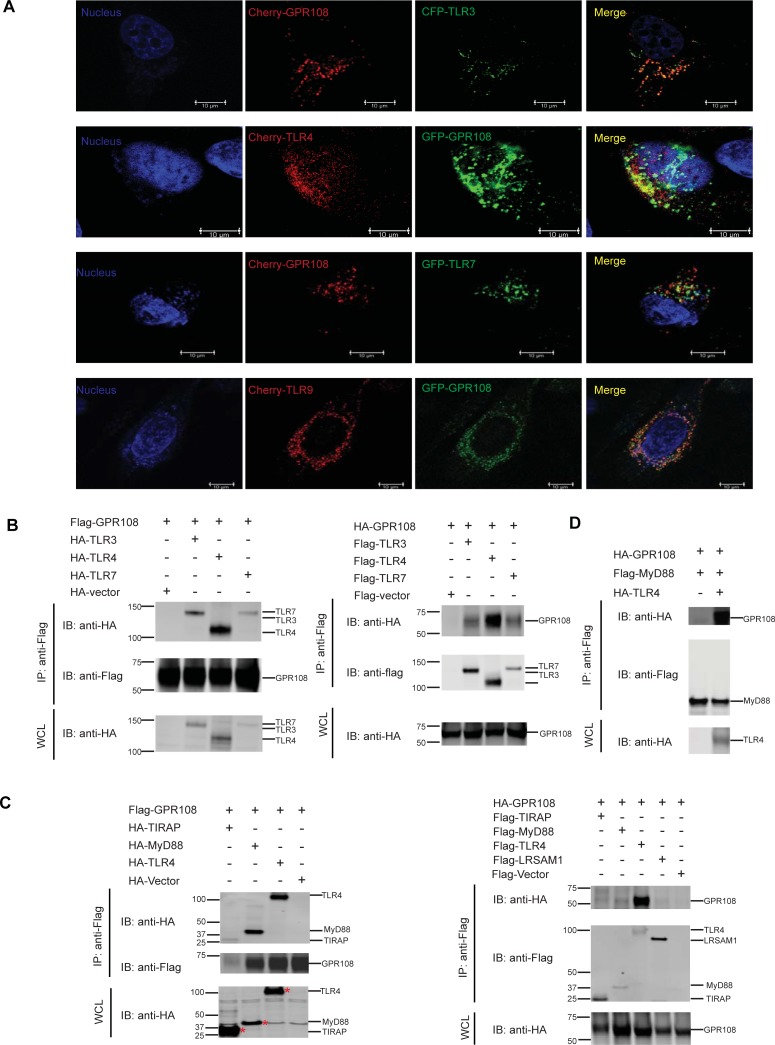
GPR108 interacted with TLRs and their partners. (A) Confocal microscopy analysis of colocalization of GPR108 and TLRs in Hela cells transfected with mCherry-GPR108 (red) with CFP-TLR3 or EGFP-TLR7 (green), or EGFP-GPR108 (green) with mCherry-TLR4 or TLR9 (red). Nuclei were stained by Hochest (blue). Scale bars, 10μm. (B) Flag or HA tagged GPR108 was co-transfected with HA or Flag tagged TLRs (TLR3, TLR4 and TLR7) in HEK293 cells. Flag tagged proteins were immunoprecipitated with M2 anti-flag beads. Both whole cell lysates (WCL) and immunoprecipitates (IP) samples were subjected for immunoblotting with anti-flag and anti-HA antibodies. (C) Flag or HA tagged GPR108 was co-transfected with HA tagged or Flag tagged adaptor proteins (TIRAP and MyD88). Flag or HA tagged TLR4 was used as positive control and LRSAM as negative control. Cells were immunoprecipitated with M2 beads and both whole cell lysates and IP samples were subjected for blotting with anti-Flag and anti-HA antibodies. (D) Flag-MyD88 and HA-GPR108 were co-transfected with or without HA-TLR4 in HEK293 cells. Cells were immunoprecipitated with M2 beads and both whole cell lysates and IP samples were subjected for blotting with anti-Flag and anti-HA antibodies. All experiments were repeated for at least three times, and the data were the representative of similar results. Left margin listed the molecular size in kilodaltons (kD).

GPR108 localization was studied by confocal microscopy of cells expressing GPR108 fused to EGFP. A high degree of colocalization was seen between GPR108 and the *cis* Golgi markers, Giantin and GM130, distributed in a perinuclear localization pattern (Panel A in S3 Fig). A much less concordant co-staining was seen with the lysosomal and mitochondrial markers (lyso-tracker and mito-tracker) (Panel B in S3 Fig). Following transient coexpression of GPR108 tagged with mCherry and TLR3 tagged with CFP, or TLR7 tagged with EGFP, or of GPR108 tagged with EGFP and TLR4 or TLR9 tagged with mCherry, extensive colocalization between GPR108 and TLRs was observed by confocal microscopy (Fig 4A).

Co-immunoprecipitation (co-IP) of proteins expressed in 293 cells was used to test if GPR108 could directly interact with TLRs. The results showed that GPR108 could bind to TLR3, 4 and 7 which was validated by reciprocal co-IP (Fig 4B). None of the GPR108 mutants lost the property of binding to TLRs (Panel C and D in S3 Fig).

TLRs act through TIR domain-contained adaptor proteins to mediate a downstream signal cascade [22–24]. A strong binding of GPR108 to MyD88 or TIRAP was observed when using GPR108 as bait in co-IP. However, only weak binding to GPR108 was found when using MyD88 or TIRAP as baits (Fig 4C). GPR108 did not bind to control protein LRSAM1 (Fig 4C). However, stronger binding of MyD88 to GPR08 was detected if TLR4 was co-expressed (Fig 4D).

### GPR108 suppresses signaling by modulating MyD88 during stimulation

**Fig 5:**
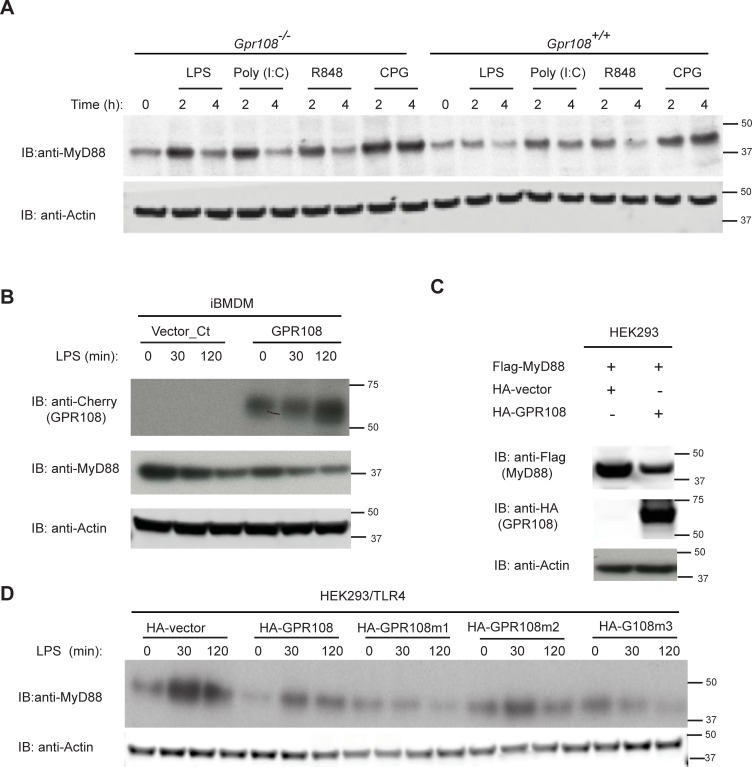
GPR108 suppresses signaling through modulating MyD88 during stimulation. (A) The enhanced MyD88 signals in primary *Gpr108*^*-/-*^ macrophages compared to that in *Gpr108*^*+/+*^ macrophages when stimulated with LPS (100 ng/ml), Poly (I:C) (5 μg/ml), R484 (10 μg/ml) and CPG (1μM) at different time points. (B) Immunoblotting analysis of MyD88 in immortalized macrophages transduced with vector control or GPR108 cDNA tagged with cherry after LPS stimulation at different time points. (C) HEK293 cells were co-transfected with Flag-MyD88 and HA-vector or HA-tagged GPR108. MyD88 and GPR108 were measured by immunoblotting with anti-HA and anti-Flag antibodies. (D) HEK293 cells were co-transfected with Flag-TLR4 and HA-vector or GPR108 or GPR108 mutant m1, m2 and m3. Endogenous MyD88 were measured by immunoblotting after stimulation with LPS at different time points. All experiments were repeated for at least three times, and the data were the representative of similar results. Right lane listed the molecular size in kilodaltons (kD).

An enhanced signal attributable to adaptor protein MyD88 was observed in primary macrophage cells derived from *Gpr108*-null mice treated with different TLR agonists, indicating a possible inhibitory effect of GPR108 on MyD88 (Fig 5A). When GPR108 was transiently expressed in iBMDM, upregulation of GPR108 was observed concomitant with the overall inhibition of MyD88 upon LPS stimulation (Fig 5B).

To elucidate their relationship, MyD88 expression was investigated in 293 cells. Both exogenously and endogenously expressed MyD88 were antagonized by expression of GPR108 (Fig 5B and 5C). GPR108 mutants m1 (extracellular domain deletion) and m3 (intracellular domain deletion) but not m2 (internal loop 3 replacement) had little or no effect on MyD88 (Fig 5D). Moreover, GPR108 and mutant m1, m2 and m3 showed different responses to LPS stimulation (Fig 5D). Mutant m2 differed little from wild-type in its effects on MyD88 during stimulation but m1 and m3 gave a different pattern from m2 mutants, suggesting an important role for the N-terminal and C-terminal domains of GPR108 in signal transduction following stimulation (Fig 5D).

### GPR108 halts the alteration of MyD88’s binding on TLR4 during LPS stimulation through regulating TRAF6-mediated MyD88 ubiquitination

**Fig 6:**
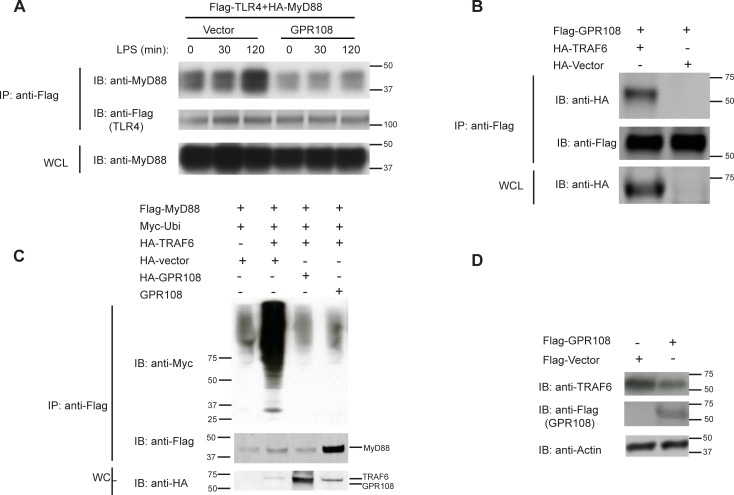
GPR108 reduces MyD88 binding on TLR4 which might be attributable to its ability of blocking MyD88 ubiquitination through negatively regulating E3 ligase TRAF6. (**A**) HEK293T cells were co-transfected with Flag-TLR4 and HA-MyD88 with or without GPR108. Cells stimulated with LPS at different time points were subjected for immunoprecipitated with M2 anti-flag beads. IP samples were blotted with anti-MyD88 and anti-flag antibodies and whole cell lysates were blotted with anti-MyD88. (B) HEK293T cells were transfected with Flag-GPR108 as well as HA-TRAF6 or HA-vector. Cells were immunoprecipitated and subjected for blotting with anti-HA and anti-Flag antibodies. (C) HEK293T cells were co-transfected with Flag-MyD88 and Myc-Ubiquitin as well as HA vector or HA-GPR108. Cells were subjected for IP and immunoblotting analysis for anti-Myc, anti-Flag and anti-HA antibodies. (D) HEK293 cells were transfected with HA-vector or GPR108-HA. Cells were subjected for blotting with anti-HA, TRAF6 and actin antibodies.

Upon activation, the formation of the TLR4-Mal-MyD88 complex contributes to NF-κB activation. The binding of MyD88 to TLR4 in the presence of GPR108 was investigated following LPS stimulation. Compared to the rapid escalation of the binding signal in short time of stimulation in the absence of GPR108, the change of MyD88’s binding on TLR4 was attenuated when GPR108 was present (Fig 6A). Co-immunoprecipitation results had shown that MyD88 bound well to GPR108 in the presence of TLR4.

Co-IP data showed that GPR108 could interact with the E3 ligase TRAF6 (Fig 6B). TRAF6 may mediate K63-linked ubiquitination of MyD88 which promotes activation [25]. To investigate if GPR108, MyD88, TRAF6 are connected, TRAF6 was co-expressed with MyD88. The level of MyD88 ubiquitination was greatly elevated following co-expression with TRAF6 and co-expression with GPR108 blocked the ubiquitination (Fig 6C). In addition, the expression of GPR108 reduced endogenous TRAF6 (Fig 6D). Hence GPR108 may block MyD88 ubiquitination by regulating TRAF6 and the resulting change in MyD88 modification may alter its affinity for GPR108 and TLR4.

### TIRAP regulates GPR108 and feedbacks to TRAF6

**Fig 7:**
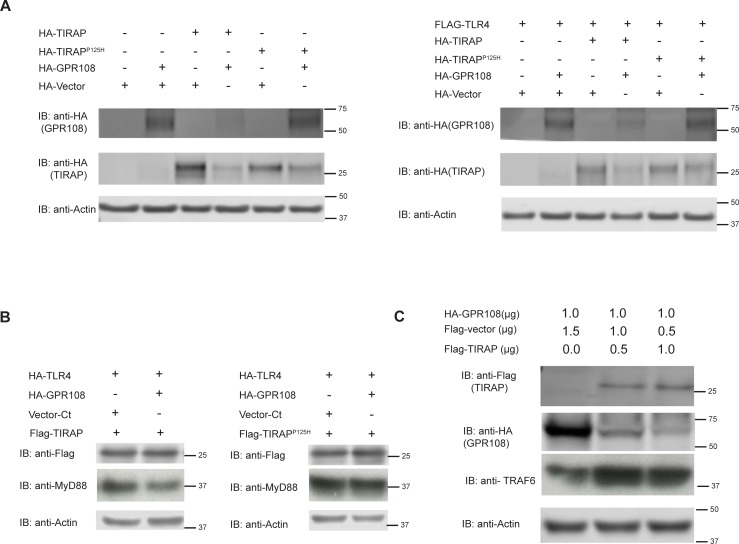
GPR108 was negatively regulated by TIRAP which feedback on TRAF6 in turn. (A) Immunoblotting analysis of HEK293T cells transfected with HA tagged TIRAP, TIRAP^P125H^ and GPR108 without flag-TLR4 or with flag-TLR4. Whole cell lysates were blotting with anti-HA antibody. (B) Immunoblotting analysis of HEK293T cells transfected with HA tagged TIRAP, TIRAP^P125H^ and GPR108 with flag-TLR4. Whole cell lysates were blotted with antibodies anti-flag as well as anti-MyD88 with actin as an internal control. (C) HEK293T cells were transfected with HA-GPR108 and Flag vector or TIRAP at different DNA amount. Cell lysates were subjected for immunoblotting analysis against Flag, HA and TRAF6. All experiments were repeated for at least three times, and the data were the representative of similar results.

TIRAP is an adaptor protein between TLRs and MyD88 [26]. The data above indicates that GPR108 can engage TLR and MyD88 and that GPR108 negatively regulates MyD88. To investigate the role of TIRAP, GPR108 was co-expressed with TIRAP and a dominant negative mutant, TIRAP^P125H^, in 293 cells. The blotting data showed whether TLR4 was co-expressed or not, wild-type TIRAP could inhibit the expression of GPR108 but TIRAP^P125H^ could not (Fig 7A). To further characterize the relationship between GPR108 and TIRAP, TLR4-HA and TIRAP-flag were co-expressed together with GPR108-HA. The results showed that MyD88 expression decreased when GPR108 was co-expressed with TIRAP, but not with TIRAP^P125H^ (Fig 7B). To investigate the relationship between GPR108 and TIRAP, TIRAP expression was titrated by introducing different amounts of expression vector in 293 cells. As the TIRAP expression increased, theGPR108 expression decreased and the TRAF6 expression increased (Fig 7C).

## Discussion

Although *Gpr108* bears structural similarity to G-protein coupled receptors, to date no study has demonstrated a functional role for the protein. The related gene *Gpr107* participates in the regulation of receptor-mediated endocytosis [10]. In this study *Gpr108*-null mice did not show any gross phenotype whereas *Gpr107*-null mice exhibited embryonic lethality.

The transcriptional profile of *Gpr108*-null MEF revealed elevated transcript abundance of TLR genes and genes associated with responses to TLR agonists in BMDM cells. Enhanced TLR signaling was observed in *Gpr108*-null MEF, BMDM cells, and in *Gpr108* deficient THP1 (human monocytoid) cells as well which were generated by Crispr/Cas9-mediated gene targeting, even though there are some discrepancy on cytokine secretion in different types of cells possibly due to different downstream regulation of signaling in those cells. Considering these findings GPR108 was expected to act as general immune suppressor and a negative regulator of TLR signaling *in vivo*. However, the tremendous efforts to further explore the role of GPR108 in whole animal level were not very successful. Although LPS lethal challenge showed more death in *Gpr108*^-/-^ mice, there was only slight difference observed in serum IL-6 level. Other tested cytokines in the panel are not different. The results can be explained that GPR108 could negatively regulate TLR signaling in certain specific cells, but its regulation will be hidden by other redundant regulators in whole animal level.

The data from *Gpr108*-null cells that GPR108 deficiency causes a constitutive inflammatory status in *Gpr108*-null cells and enhanced TLR-triggered immune response implies its role as a negative regulator. However, transient overexpression of GPR108 in 293 cells strongly increased NF-κB and IFNβ reporter activity and restricted the TLR-triggered response, implying its dual regulations acting as an activator and a suppressor. Stable reconstitution of GPR108 in *Gpr108*-null MEF, iBMDM and THP-1 could not be accomplished. To reconstitute GPR108, inducible GPR108 expression was introduced into *Gpr108*-null iBMDM. Surviving cells could only be induced by DOX at a very low level to weakly activate NF-κB reporter but even at low levels restricted TLR-triggered NF-κB activation and cytokine production, consistent with the hypothesis that GPR108 acts as a negative regulator of TLR signaling and as an NF-κB activator. It also tells us that it is possible that GPR108 expression must be restrained to avoid a toxic activation, possibly involving NF-κB and/or other pathways. So physiologically it dominantly works as a negative regulator due to its low level of expression in natural cells.

TLR-mediated signaling is initiated by ligand binding and assembly of a complex of adaptors and kinases to transduce downstream signal cascades [4, 5]. Where does GPR108 fit in this network? Although GPR108 results in NF-κB activation, it also inhibits NF-κB reporters when co-expressed with TLRs and relevant downstream proteins like MyD88, TIRAP, IRAK, TRAF6 and Nemo. MyD88 is a common adaptor for all TLRs, except TLR3[19]. MyD88 contains a TIR domain, which mediates interaction with the TIR of TLRs. GPR108 directly engages TLRs and partners MyD88, TIRAP and TRAF6. GPR108 regulates the abundance of MyD88 during stimulation. Less MyD88 protein and less MyD88 binding to TLR4 upon stimulation were found when GPR108 was overexpressed. These results are consistent with the view that GPR108 may exhibit dual functionality during signal transduction. Under normal circumstances GPR108 acts as a negative regulator to avoid excessive activation. However, under special circumstances, possibly when a pathogen protein interferes with the formation of a TLR-dependent signaling complex, GPR108 may become liberated from its suppressive context and directly contribute an alarm signal that may in many cases result in cell death.

In previous studies TRAF6 and Cbl-B have been identified as E3 ligases that may regulate MyD88 [27, 28]. The present data indicate TRAF6 increases MyD88 ubiquitination and that GPR108 expression blocks the ubiquitination. Co-IP data show that TRAF6 is a direct GPR108 binding partner, and that GPR108 expression inhibits TRAF6 expression. Hence GPR108 may reduce MyD88 signaling by interfering with its modification by TRAF6. The reciprocal effects of GP108 and MyD88 on binding to TLR4 suggest that GPR108 and TLR4 may compete to bind MyD88. K63-linked ubiquitination like that seen on MyD88 is usually associated with immune signaling activation [25].

**Fig 8:**
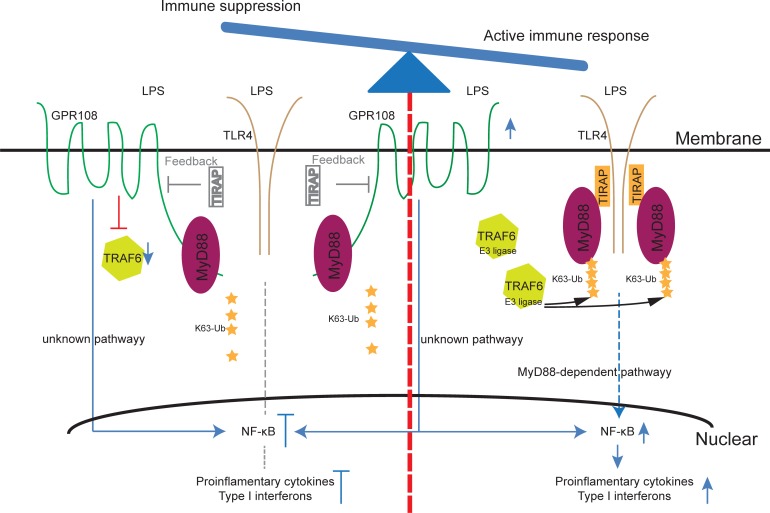
GPR108 works as a dual functional regulator to balance the TLR-triggered immune response. For active immune response to defend the invading pathogens, TRAF6 mediates K63-linked MyD88 ubiquitination conferring its higher binding affinity to TLR4-TIRAP and inducing the downstream signaling activation through MyD88-dependent pathway (right side). At the same time, GPR108 expression is going up upon stimulation. It may mediate some unknown activation pathway in synergy with the TLR-triggered signaling. However, when signaling goes up higher, the elevated GPR108 would damp TRAF6 and reduce MyD88’s ubiquitination level which attenuates MyD88’s binding to TLR4 and collapses downstream signaling. So GPR108 suppresses the signaling by binding the de-ubiquitinated MyD88 that might possess higher affinity to GPR108 than TLR4 (left side). This regulatory loop will keep the signal activation within the appropriate range. For another level of counter regulation, TIRAP could restrict GPR108 which might act as an activator through an unknown independent pathway.

Based on the above data, a model can be proposed (Fig 8). Upon LPS stimulation, TRAF6 increases K63-linked MyD88 ubiquitination, increasing its affinity for TLR4 and inducing downstream signaling. GPR108 expression rises with stimulation, possibly as an adaptive response to prevent sustained high engagement. The elevated GPR108 reduces TRAF6 and reduces MyD88 ubiquitination which attenuates MyD88 binding to TLR4 and reduces downstream signaling.

Although GPR108 is a negative regulator of TLR-triggered immune responses, GPR108 is also a very strong activator when overexpressed and is up-regulated upon LPS stimulation. Stable overexpression of GPR108 is difficult to achieve and elevated expression of GPR108 appears to be detrimental to cells. TIRAP was found to be a suppressor of GPR108 expression in this study. Titration of TIRAP in 293 cells led to decreasing GPR108 but increasing TRAF6. TIRAP is an adaptor protein engaging TLR4 and MyD88 [26]. The mutant TIRAP^P125H^ has a dominant negative effect on TLR4 signaling [26]. TIRAP^P125H^ does not affect expression of GPR108. It is possible that TIRAP^P125H^ cannot efficiently engage TLR and MyD88 and so regulation of GPR108 on TLR4 and MyD88 will be interfered.

Domain mutagenesis revealed that the intracellular loop3 and C-terminal domain of GPR108 contribute to negative regulation of MyD88. Deletion of the N- or C-terminal domain of GPR108 disrupted the GPR108 effect on the response to LPS. It was observed that GPR108 enhanced its binding to MyD88 when TLR4 was introduced, indicating that there might be a dynamic configuration change for TLR4 and GPR108 during TLR-triggered immune responses. The present study showed that GPR108 negatively regulated TLR-triggered immune responses through antagonizing MyD88 but also worked as an activator suppressed by TIRAP. Hence GPR108 may play a critical role in balancing stimulatory and inhibitory TLR-triggered immune responses.

## Supporting information

S1 Fig mRNA levels of *Gpr108* in different mouse tissues. (A, B) mRNA abundance of *Gpr108* were tested by RT-MLPA using two pairs of probes located at the beginning and terminal region. (C) mRNA abundance of *Gpr108* were tested by RT-qPCR. (D)The top up-regulated mRNAs in *Gpr108*-null BMDM cells compared to that in wildtype BMDM cells derived from mice (n = 3) in the absence or presence of different TLR agonists. Each column presents the mRNA expression of BMDM cells without or with different treatments. Around 50 immune response and immediate early genes (IEGs) were selected (right side) and shown in Fig 1F. The intensity represents the magnitude of the difference. Red and green denote low and high expression, respectively.
(PDF)

S2 Fig Generation of *Gpr108*^-/-^ THP-1 cells and cytokine measurements in *Gpr108*^-/-^ cells and mice. (A) *Gpr108*^-/-^ THP-1 cell clones were identified by amplifying the region flanked by a pair of gRNAs. E5 and G12 clones were screened out containing the deletion. mRNA abundance of GPR108 in clone E5 and G12 were lost compared to wild-type cells by RT-qPCR measurement. (B) TNFα and IL-1β expression were dramatically increased in *Gpr108*^-/-^ THP-1 G12 clone treated with LPS for 18hours. (C) Survival curve of *Gp108*^+/+^ (n = 14) and *Gpr108*^-/-^ (n = 13) mice, monitored every 2 hours after lethal challenge with LPS (10μg/kg) and L-galactosamine (800 mg/kg). (*P<0.05 Wilcoxon test). (D) The serum IL-6 level after 1.5 hours of injection.
(PDF)

S3 Fig Colocalization test of GPR108 with various cellular organelles and interaction of GPR108 and its mutants with TLR3, 4, 7 and 9. (A) Great colocalization of GPR108 with Golgi marker Giantin and GM130. (B) Less co-staining signals were observed with Lyso-tracker and Mito-tracker. (C) HEK293 cells were cotransfected with TLR3 or 4-flag and GPR108-HA, or GPR108 mutant m1 or m2 or m3-HA. Flag-tagged TLR3 or 4 was immunoprecipitated with anti-Flag beads and blotted with anti-HA. (D) HEK293 cells were cotransfected with TLR7 or 9-flag and GPR108-HA, or GPR108 mutant m1 or m2 or m3-HA. Flag-tagged TLR3 or 4 was immunoprecipitated with anti-Flag beads and blotted with anti-HA.
(PDF)

S1 Table Sequence for MLPA probes and genotyping primers of *Gpr108*-null mice. (DOCX)

S2 Table Sequence for RT-qPCR primers. (DOCX)

S3 Table Sequence for RT-MLPA probes. (DOCX)

S4 Table The gRNA Sequences and identification primers for generating *GPR108*-null THP1 cells by Crispr/Cas9 strategy. (DOCX)

S5 Table List of toll-like receptor genes upregulated in *Gpr108*-null MEF cells. (DOCX)
