# Supplementary figures and images for: GPR108, an NF-κB activator suppressed by TIRAP, negatively regulates TLR-triggered immune responses
Source: PLoS One. 2018 Oct 17;13(10):e0205303. doi: 10.1371/journal.pone.0205303 (PMC6192633; doi:10.1371/journal.pone.0205303)

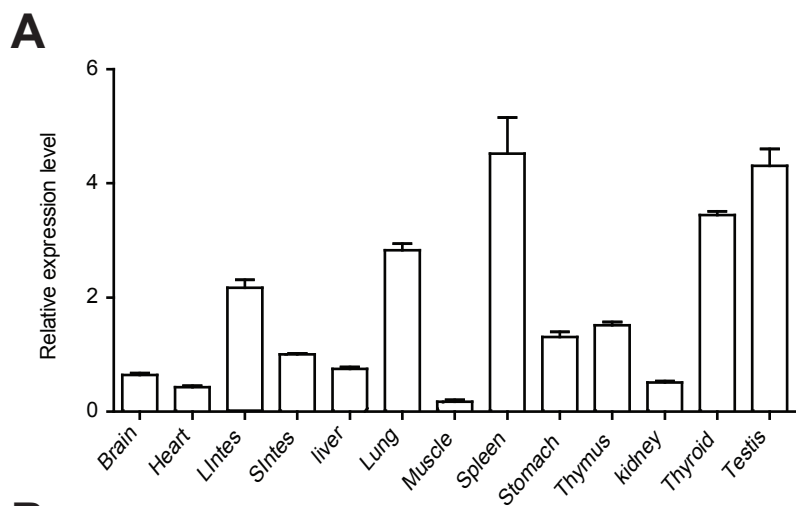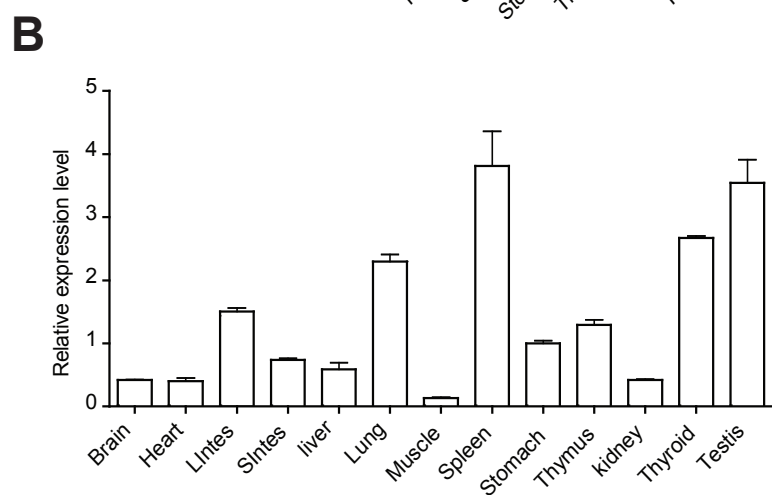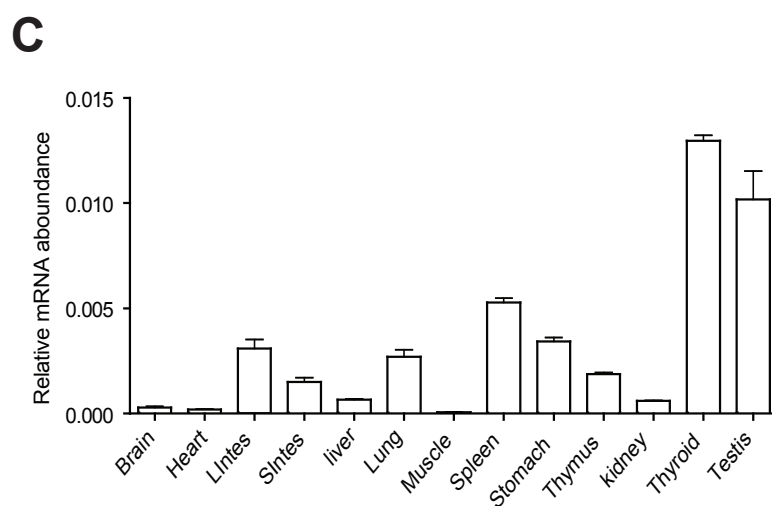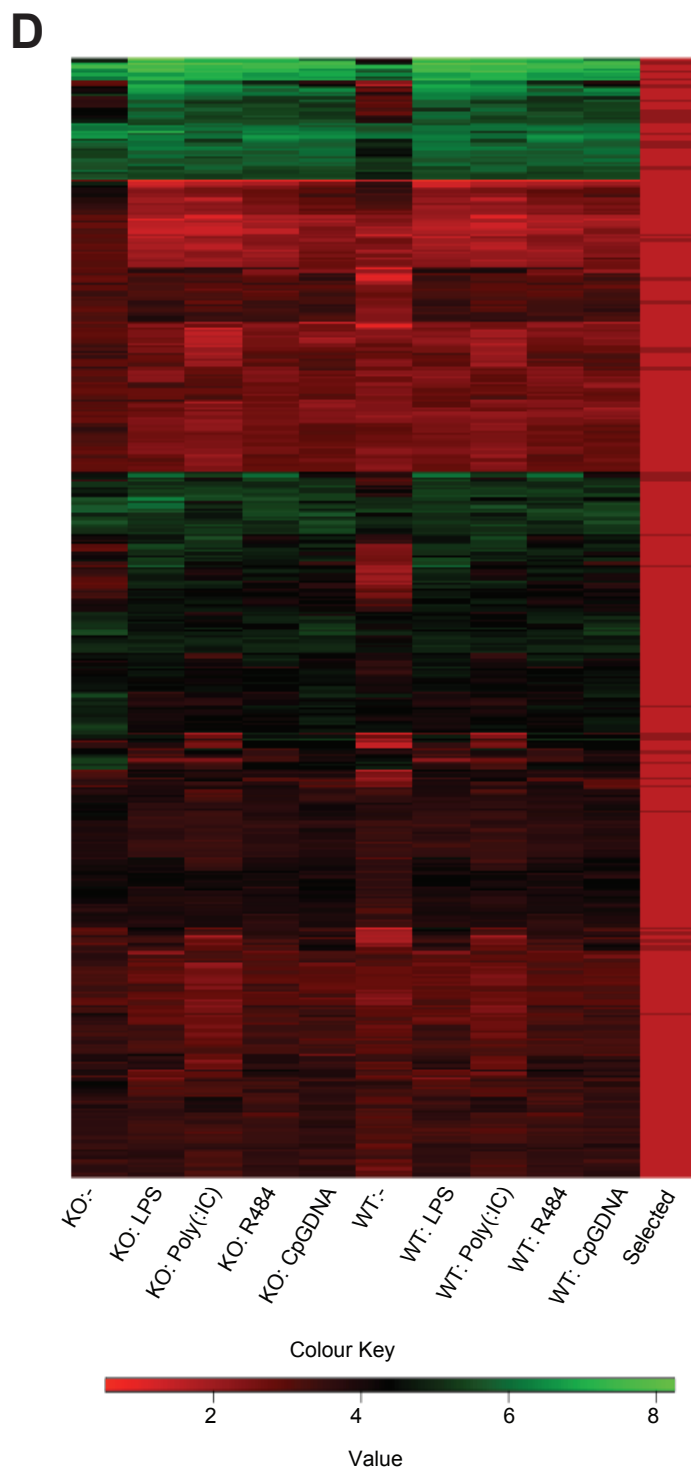

Supplement: S1 Fig — (A, B) mRNA abundance of Gpr108 were tested by RT-MLPA using two pairs of probes located at the beginning and terminal region. (C) mRNA abundance of Gpr108 were tested by RT-qPCR. (D)The top up-regulated mRNAs in Gpr108-null BMDM cells compared to that in wildtype BMDM cells derived from mice (n = 3) in the absence or presence of different TLR agonists. Each column presents the mRNA expression of BMDM cells without or with different treatments. Around 50 immune response and immediate early genes (IEGs) were selected (right side) and shown in Fig 1F. The intensity represents the magnitude of the difference. Red and green denote low and high expression, respectively. (PDF) [file pone.0205303.s001.pdf]

**A**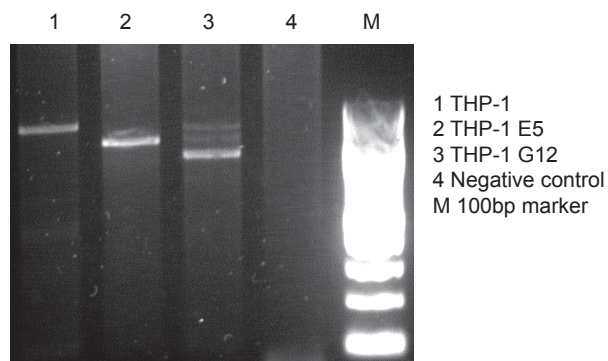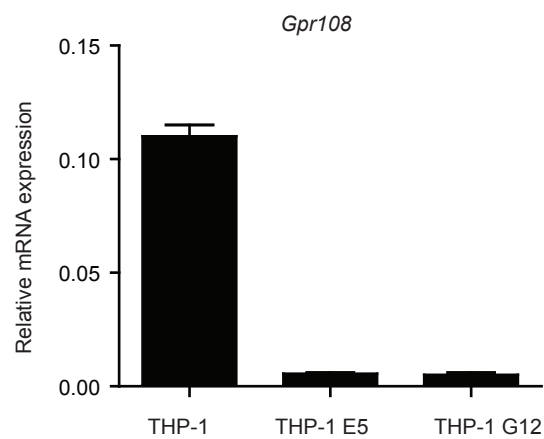**B**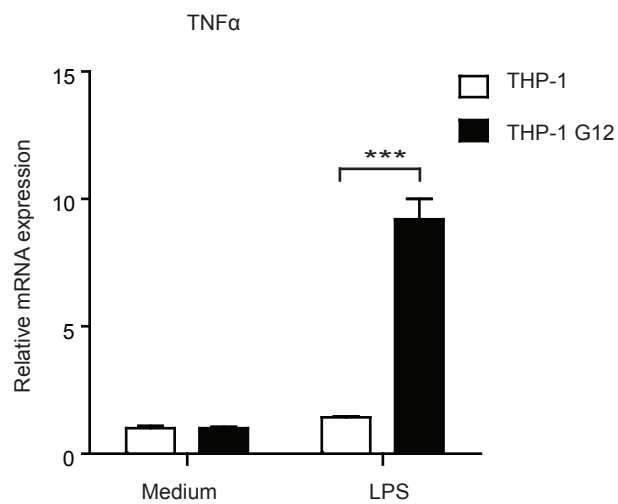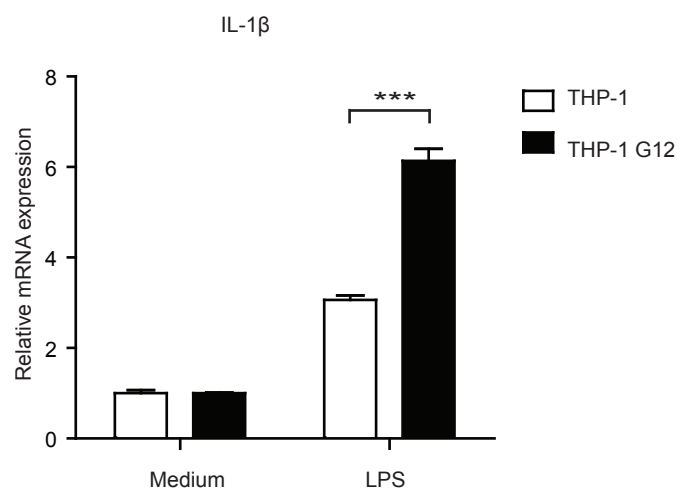**C**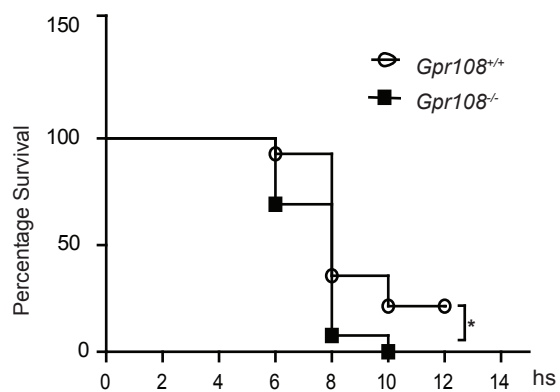**D**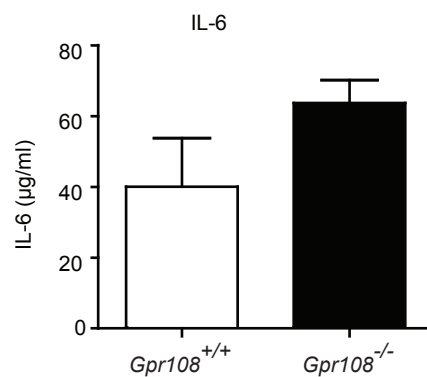

Supplement: S2 Fig — (A) Gpr108-/- THP-1 cell clones were identified by amplifying the region flanked by a pair of gRNAs. E5 and G12 clones were screened out containing the deletion. mRNA abundance of GPR108 in clone E5 and G12 were lost compared to wild-type cells by RT-qPCR measurement. (B) TNFα and IL-1β expression were dramatically increased in Gpr108-/- THP-1 G12 clone treated with LPS for 18hours. (C) Survival curve of Gp108+/+ (n = 14) and Gpr108-/- (n = 13) mice, monitored every 2 hours after lethal challenge with LPS (10μg/kg) and L-galactosamine (800 mg/kg). (*P<0.05 Wilcoxon test). (D) The serum IL-6 level after 1.5 hours of injection. (PDF) [file pone.0205303.s002.pdf]

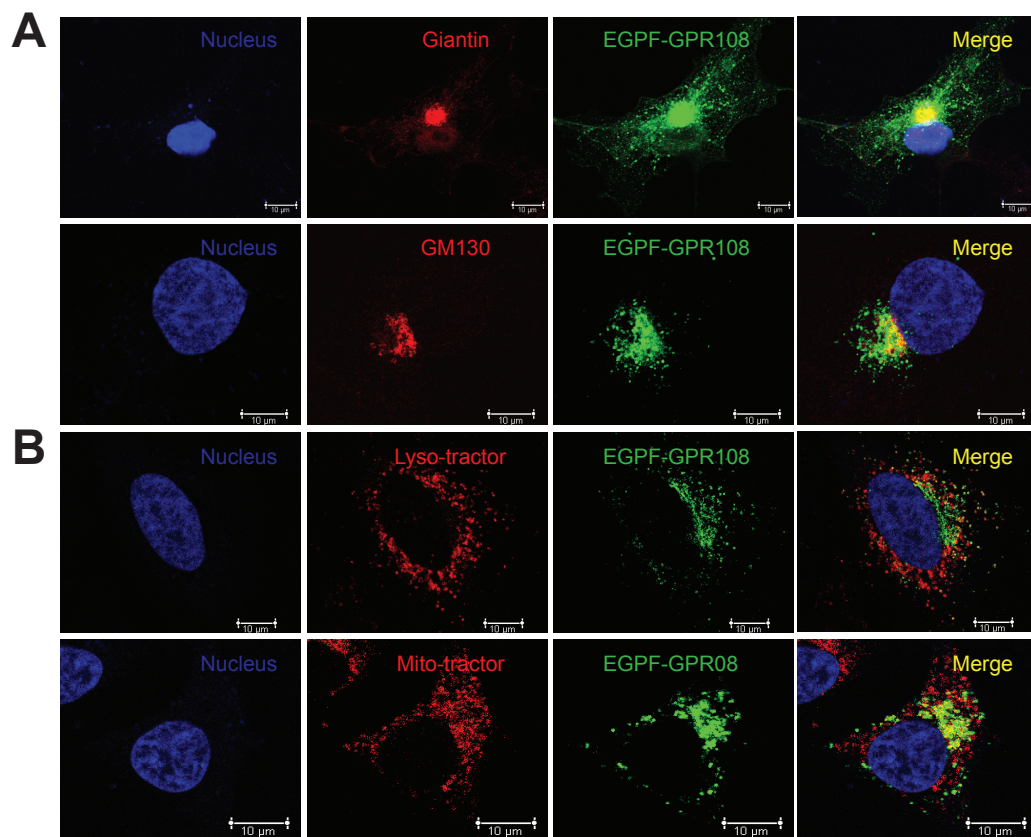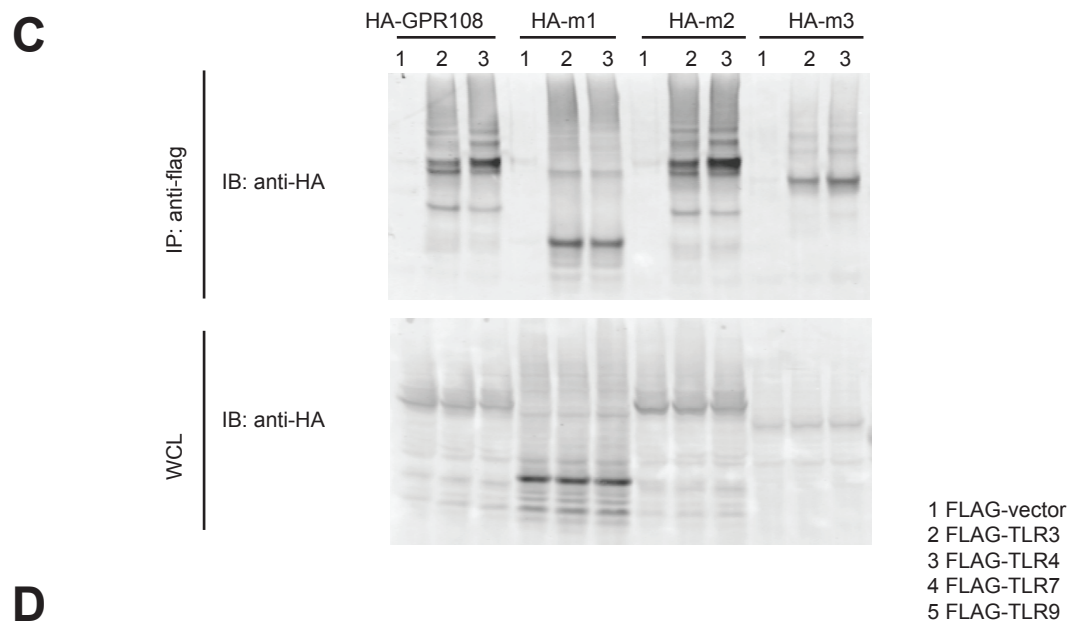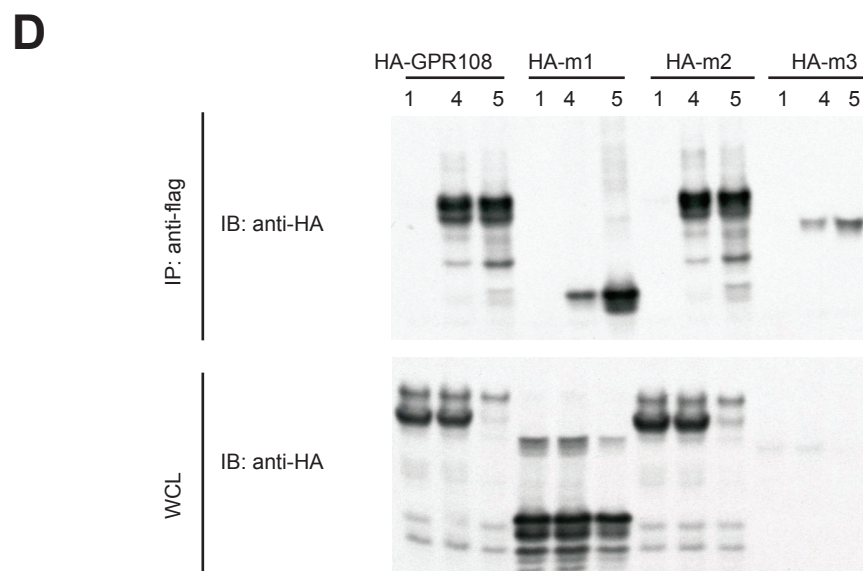

Supplement: S3 Fig — (A) Great colocalization of GPR108 with Golgi marker Giantin and GM130. (B) Less co-staining signals were observed with Lyso-tracker and Mito-tracker. (C) HEK293 cells were cotransfected with TLR3 or 4-flag and GPR108-HA, or GPR108 mutant m1 or m2 or m3-HA. Flag-tagged TLR3 or 4 was immunoprecipitated with anti-Flag beads and blotted with anti-HA. (D) HEK293 cells were cotransfected with TLR7 or 9-flag and GPR108-HA, or GPR108 mutant m1 or m2 or m3-HA. Flag-tagged TLR3 or 4 was immunoprecipitated with anti-Flag beads and blotted with anti-HA. (PDF) [file pone.0205303.s003.pdf]
