# Supplementary material for: GPR108, an NF-κB activator suppressed by TIRAP, negatively regulates TLR-triggered immune responses
Source: PLoS One. 2018 Oct 17;13(10):e0205303. doi: 10.1371/journal.pone.0205303 (PMC6192633; doi:10.1371/journal.pone.0205303)
Supplement: S1 Table — (DOCX) [file pone.0205303.s004.docx]

| Product | Name | Sequence | size |
| --- | --- | --- | --- |
| 5WT | P1-Lig | GGGTTCCCTAAGGGTTGGAGTAGGGGTGCGAGGTGCAGGAGGTCTG | 107 |
|  | P2-NPK | AGGGCGGCTCACTGGACACAGGGAAGTCACAACAGCTCCCTAGATTGGATCTTGCTGGCGC |  |
| 5mt | P5-NPK | AGGGCCGTCTCTGGAGGGCGCGCCGGGAGTAGTGCCTAGATTGGATCTTGCTGGCGC | 103 |
| 3WT | P3-Lig | GGGTTCCCTAAGGGTTGGAGGCTGCGACTGCCCCCCGAACACAGCCTCT | 111 |
|  | P4-NPK | GACGCCTGATAAGGCCTACCCACTTTTCAGCCAATAGTCGCCTAGATTGGATCTTGCTGGCGC |  |
| 3mt | P6-Lig | GGGTTCCCTAAGGGTTGGAGTACTGCTTGTGGGTACACTCTGCGGGTGGGATCCGTCTCTCTCG | 127 |
| Neo | P7-Lig | GGGTTCCCTAAGGGTTGGAGCTATTCGGCTATGACTGGGCACAACAGACAATCGGCT | 116 |
|  | P8-NPK | GCTCTGATGCCGCCGTGTTCCGGCTGTCAGCGCAGGCCTAGATTGGATCTTGCTGGCGC |  |
| C1 | P9-Lig | GGGTTCCCTAAGGGTTGGACATCTCCTTTTGCTTCAGGGGTGTACACCTTAAACGACGAGAAG | 122 |
|  | P10-NPK | CAATGGGTGAACACAGTCGCTGGAGAGAAACTCCCCCCTAGATTGGATCTTGCTGGCGC |  |
| C2 | P11-Lig | GGGTTCCCTAAGGGTTGGACATCATGGCTTCTGCTAAGATGACCTCTCGTCTCCCTCCAGTATGAAGAA | 137 |
|  | P12-NPK | TGCCTGCTTGCCCATGTTTGGCTACAAACACGTGCTGACGCTAACCCCTAGATTGGATCTTGCTGGCGC |  |
| P1/P2 | P1 | TGAGATTGGGAGGAGTTTGG | 621 |
|  | P2 | CTGCCTCGGACTGAAGTAGG |  |
| P3/P4 | P3 | TGAGATTGGGAGGAGTTTGG | 362 |
|  | P4 | CTGCCTCGGACTGAAGTAGG |  |
