# Supplementary material for: GPR108, an NF-κB activator suppressed by TIRAP, negatively regulates TLR-triggered immune responses
Source: PLoS One. 2018 Oct 17;13(10):e0205303. doi: 10.1371/journal.pone.0205303 (PMC6192633; doi:10.1371/journal.pone.0205303)
Supplement: S2 Table — (DOCX) [file pone.0205303.s005.docx]

| Product | Sequence | Size (bp) |
| --- | --- | --- |
| Actin | GGCTGTATTCCCCTCCATCG | 154 |
|  | CCAGTTGGTAACAATGCCATGT |  |
| GAPDH | AGGTCGGTGTGAACGGATTTG | 123 |
|  | TGTAGACCATGTAGTTGAGGTCA |  |
| mIFNβ | AGCTCCAGCTCCAAGAAAGGACGAACAT | 87 |
|  | GCCCTGTAGGTGAGGTTGATCT |  |
| mIL-1β | TGCCACCTTTTGACAGTGATG | 138 |
|  | TGATGTGCTGCTGCGAGATT |  |
| hIL-1β | ACCTGTCCTGCGTGTTGAAA | 122 |
|  | GGGGAGAAGGTGGTTGTCTG |  |
| mIL-6 | TAGTCCTTCCTACCCCAATTTCC | 76 |
|  | TTGGTCCTTAGCCACTCCTTC |  |
| mTNFα | ACTGATGAGAGGGAGGCCAT | 149 |
|  | CCGTGGGTTGGACAGATGAA |  |
| hTNFα | TGAAAGCATGATCCGGGACG | 189 |
|  | CAGGCACTCACCTCTTCCCT |  |
| mGPR108 | CGTCATTCACCCATCTCCTACA | 93 |
|  | TGTTCCCCTGGGGTAGTTTTAT |  |
| hGPR108-1 | CGTGGTGATCGGCTCTCAG | 95 |
|  | TGTCGAATGGATGCTCCTTTC |  |
| hGPR108-2 | CTTCCCGGAACCCAGAGTCCGT | 115 |
|  | CTCACGGGCTACAAGTTCCAGC |  |
