# Supplementary material for: GPR108, an NF-κB activator suppressed by TIRAP, negatively regulates TLR-triggered immune responses
Source: PLoS One. 2018 Oct 17;13(10):e0205303. doi: 10.1371/journal.pone.0205303 (PMC6192633; doi:10.1371/journal.pone.0205303)
Supplement: S3 Table — (DOCX) [file pone.0205303.s006.docx]

| Product | Sequence | Size (bp) |
| --- | --- | --- |
| GAPDH-Lig | GGGTTCCCTAAGGGTTGGAGGCCAAGGTCATCCATGACAACTTTGGCATTGTGGAAGGGCTCATG | 135 |
| GAPDH-NPK | ACCACAGTCCATGCCATCACTGCCACCCAGAAGACTGTGGATGGCCCCCTAGATTGGATCTTGCTGGCGC |  |
| ACTB-Lig | GGGTTCCCTAAGGGTTGGACTAAGGCCAACCGTGAAAAGATGACCCAG | 89 |
| ACTB-NPK | ATCATGTTTGAGACCTTCAACACCCCAGCCCTAGATTGGATCTTGCTGGCGC |  |
| HPRT1_lig | GGGTTCCCTAAGGGTTGGAGGATTTGGAAAAAGTGTTTATTCCTCATGGACTGATTATGGACA | 125 |
| HPRT1_NPK | GGACTGAAAGACTTGCTCGAGATGTCATGAAGGAGATGGCCTAGATTGGATCTTGCTGGCGC |  |
| TBP_lig | GGGTTCCCTAAGGGTTGGACTTATGCTCAGGGCTTGGCCTCCCCA | 95 |
| TBP_NPK | CAGGGCGCCATGACTCCTGGAATTCCCCCTAGATTGGATCTTGCTGGCGC |  |
| Gpr108-Lig_1 | GGGTTCCCTAAGGGTTGGACTCCATCCCAGGCCAGGAGCAGCCATTCGACCTCACT | 120 |
| Gpr108-NPK_1 | GTGATGATCCGGGAGAAGAATCCAGAAGGCTTCTTGTCAGCCCTAGATTGGATCTTGCTGGCGC |  |
| Gpr108-Lig_2 | GGGTTCCCTAAGGGTTGGAGGAACACAGGCACTCATCCGAGAGACAGCCACCCACGC | 115 |
| Gpr108-NPK_2 | AGGACCCCAGTGGGAAGGAGAAGGATCAGGTGTTGCCTAGATTGGATCTTGCTGGCGC |  |
