# Supplementary material for: GPR108, an NF-κB activator suppressed by TIRAP, negatively regulates TLR-triggered immune responses
Source: PLoS One. 2018 Oct 17;13(10):e0205303. doi: 10.1371/journal.pone.0205303 (PMC6192633; doi:10.1371/journal.pone.0205303)
Supplement: S4 Table — (DOCX) [file pone.0205303.s007.docx]

| Name | Sequence |
| --- | --- |
| gRNA1 | CTCACCAGACTACGGGGAACAGG |
| gRNA2 | CATCATCATCGAGTCCCGCGAGG |
| gRNA3 | GGGCCCCGCCGTGCGGCGATTGG |
| gRNA4 | GACCCCAGACCCTCGCGCGGCGG |
| gRNA5 | GATGGAGCCTCGCCCCCGAAGG |
| gRNA6 | ATCCCCTTCGGTCCTCCGGATGG |
| gRNA7 | GGACAGCACCGGTTCCGAATGG |
| hGPR108P1 | AGGGCAGAGAGGCCAGATAA |
| hGPR108P2 | CGTGTTCCTAATCCCCCACC |
| hGPR108P3 | AGCTGTTCAGCTGGATGTCCGCTC |
| hGPR108P4 | CTCCCGCTGCATTCTGGGTAATGTAGTC |
