# Supplementary material for: GPR108, an NF-κB activator suppressed by TIRAP, negatively regulates TLR-triggered immune responses
Source: PLoS One. 2018 Oct 17;13(10):e0205303. doi: 10.1371/journal.pone.0205303 (PMC6192633; doi:10.1371/journal.pone.0205303)
Supplement: S5 Table — (DOCX) [file pone.0205303.s008.docx]

| mRNA-6 | mRNA-7 | Normalized WT | Normalized KO | Fold WT/KO | Gene Info |
| --- | --- | --- | --- | --- | --- |
| 344 | 865 | 344 | 1746 | 0.24 | toll-like receptor 13 |
| 146 | 442 | 146 | 892 | 0.25 | toll-like receptor 7 |
| 63 | 171 | 63 | 345 | 0.37 | toll-like receptor 8 |
| 398 | 576 | 398 | 1163 | 0.39 | toll-like receptor 4 |
| 581 | 694 | 581 | 1401 | 0.45 | toll-like receptor 2 |
| 81 | 127 | 81 | 256 | 0.51 | toll-like receptor 3 |
| 13 | 25 | 12 | 50 | 0.75 | toll-like receptor 1 |
| 82 | 55 | 82 | 111 | 0.86 | toll-like receptor 6 |
| 12 | 9 | 12 | 18 | 0.95 | toll-like receptor 9 |
| 20 | 10 | 20 | 20 | 1 | toll-like receptor 5 |
